# Transport accessibility and social demand: A case study of the Tibetan Plateau

**DOI:** 10.1371/journal.pone.0257028

**Published:** 2021-09-27

**Authors:** Xingchuan Gao, Dongqi Sun

**Affiliations:** Key Laboratory of Regional Sustainable Development Modeling, Institute of Geographical Sciences and Natural Resources Research, Chinese Academy of Sciences, Beijing, China; Northeastern University (Shenyang China), CHINA

## Abstract

The equity of transport accessibility is a prerequisite for sustainable development targets, especially in the ecologically fragile area of the Tibetan Plateau (also known as the Qinghai-Tibet Plateau). The relationship between transportation supply and social demand has become a key element of socioeconomic development and environmental protection in agricultural and pastoral areas. Based on data from transportation networks, permanent populations and the economy, this study uses a network analysis model, the coefficient of variation and the Gini coefficient to construct an index of social demand in townships and analyse the equity of transport accessibility on the Tibetan Plateau between 1980 and 2017; the principle of geographic distribution and the spatial relationship between transport accessibility and social demand at the township scale are also discussed. This study finds the following: the development of transportation has improved accessibility on the Tibetan Plateau, creating a highly accessible region with important cities as the nodes and major traffic arteries as the axes; both the coefficient of variation of transport accessibility and the Gini coefficient have increased slightly; and the equity of transport accessibility among townships on the Tibetan Plateau has exhibited a downward trend. Further, the social demand index is doubling every ten years, the spatial distribution has regional characteristics, and a decrease in permanent populations is the main reason for declining social demand index scores among townships. Townships with the lowest and highest social demand index scores for transportation development enjoy greater transportation benefits; there is a significant spatial relationship between social demand and location conditions (potential accessibility); the aggregation of social demand and accessibility types follows specific geographical distribution principles; and the Mangya-Gongshan Line delineates the distribution characteristics of township clusters with low social demand and low accessibility.

## Introduction

Transportation is very important to a region’s economic structure. It is not only a key factor in social cohesion [1] but also reflects regional imbalances. Providing transportation services is one of the basic contemporary functions of government. Regarding allocation, there are always issues concerning equitable accessibility [2, 3]. Transportation equity is derived from research on equity and justice in other fields [4] in which “equity” is regarded as a core meaning of “fairness” [4–6]. Transportation equity is fundamental to achieving sustainable development goals [7, 8], which can be divided into horizontal transportation equity and vertical transportation equity [9]. Horizontal transportation equity focuses on the difference in travel demand, while vertical transportation equity advocates that traffic services should be available to specific vulnerable groups [9, 10].

There is no unified concept of transportation equity [11–13], but it has a significant social and economic impact, so it concerns people’s quality of life. Todd Litman (2005) was the first to systematically analyse transportation equity [14]. He believed it to be a basic requirement of social equity. Given its ability to solve urban transportation problems, urban transportation equity has attracted the attention of an increasing number of scholars. In recent years, many studies have been conducted on urban transportation inequality, with a focus on the spatial inequality of transportation and service facilities. Achieving the fair distribution of urban public facilities is the most important goal of planners [15]. Markham and Doran (2015), for example, found that unequal service accessibility may result in racially discriminatory spatial inequalities [2]. Neutens (2015) reviewed accomplishments in modelling accessibility to health care services and considered how to improve the measurement of equity considerations in empirical research [16]. Research by Sukaryavichute and Prytherch (2018) showed that urban rapid transit planning can reshape public transportation networks and affect the efficiency and equity of transport accessibility [17]. Different groups of people in cities have also been the targets of research on transport accessibility equity. Litman (2003) pointed out that approximately one-third of Canadian families have at least one member who belongs to a group with transportation vulnerability and that an irrational allocation of transportation resources can lead to the social exclusion of these groups [9]. Xiao et al. (2017) looked at the distribution of Chinese urban park services and the issue of equity in relation to marginalised groups [18]. Yuan, Xu and Wang (2017) established a demand index for the enjoyment of parks among socially vulnerable groups to discuss the spatial equity of, and quantitatively measure, parks in Shanghai [19].

Researchers have also looked at regional accessibility and spatial equity issues brought about by the development of transportation, especially the Trans-European Transport Network and high-speed railway (HSR). A more even spatial distribution of transport accessibility means greater equity, stronger territorial cohesion and a higher degree of integration [20]. As a result, developing transportation and reducing accessibility discrepancies between regions are key to achieving the European Union’s territorial cohesion goals and regional integrated development [21]. It is also important for countries such as the United Kingdom and Poland to promote economic ties and to improve regional equity and balanced development [22–24]. González-González and Nogués (2019) believe that transportation development widened the accessibility gap between urban and rural areas in northwest Spain, but expansion of the transportation network balanced the situation, enabling more equitable access between urban and rural areas and partially realising the goal of territorial cohesion [25]. Kim and Sultana (2015) found that early HSR construction decreased spatial equity, but the continued expansion of HSR networks enhanced equitable accessibility [26]. Yan et al. (2018) analysed the temporal and spatial characteristics of transport accessibility and spatial equity in relation to HSR in China and found that a pattern of “corridors” and “islands” of HSR lines and stations is being created [27]. Research by Wang and Zhang (2019) showed that the construction of HSR brings the most significant accessibility benefits to cities with HSR stations, leading to greater regional inequality in accessibility [28]. Li, Wang and Hilmola (2020) believe that HSR has narrowed the income gap between urban and rural areas in China, but the convergence effect on the income gap is relatively weak [29]. Gao, Li and Cao (2019) analysed the distribution effect and changing spatial equity of accessibility caused by the development of transportation infrastructure on the Tibetan Plateau [30].

The issue of transportation equity is so important that Stepniak and Rosik (2013) [23], Golub and Martens (2014) [31] and Nahmiasbiran, Martens and Shiftan (2017) [32] have all proposed applying the theory of equity to the transportation policy and transportation project evaluations. Transportation policies and development should meet the social needs of different regions to reduce the economic gap or social welfare differences among regions and avoid regional imbalances [33]. As Vickerman pointed out, development without equity is not development [34]. With the rapid development of the social economy and the improvement of living standards, the traffic and social needs of different regions are also different. To a certain extent, social demand indicators depend on economic and demographic indicators [27, 35]. Theoretically, the development of transportation should be synchronized with the development of social demand. In this study, transportation equity refers to horizontal transportation equity. As such, transport accessibility is a dual issue concerning social equity and spatial equity. Transport accessibility equity requires attention to be paid not only to spatial equity but also to social equity.

In summary, the existing literature mainly focuses on the social demand and traffic accessibility in urban areas and not on the spatial equity of transportation development in non-urban areas. The questions posed by this study involve whether transport accessibility meets local social demand in non-urban areas with rapidly developing railways and highways, especially in agricultural and pastoral areas, and the spatial relationship between transport accessibility and social demand.

## Research area, data sources and research methods

### Research area

**Fig 1:**
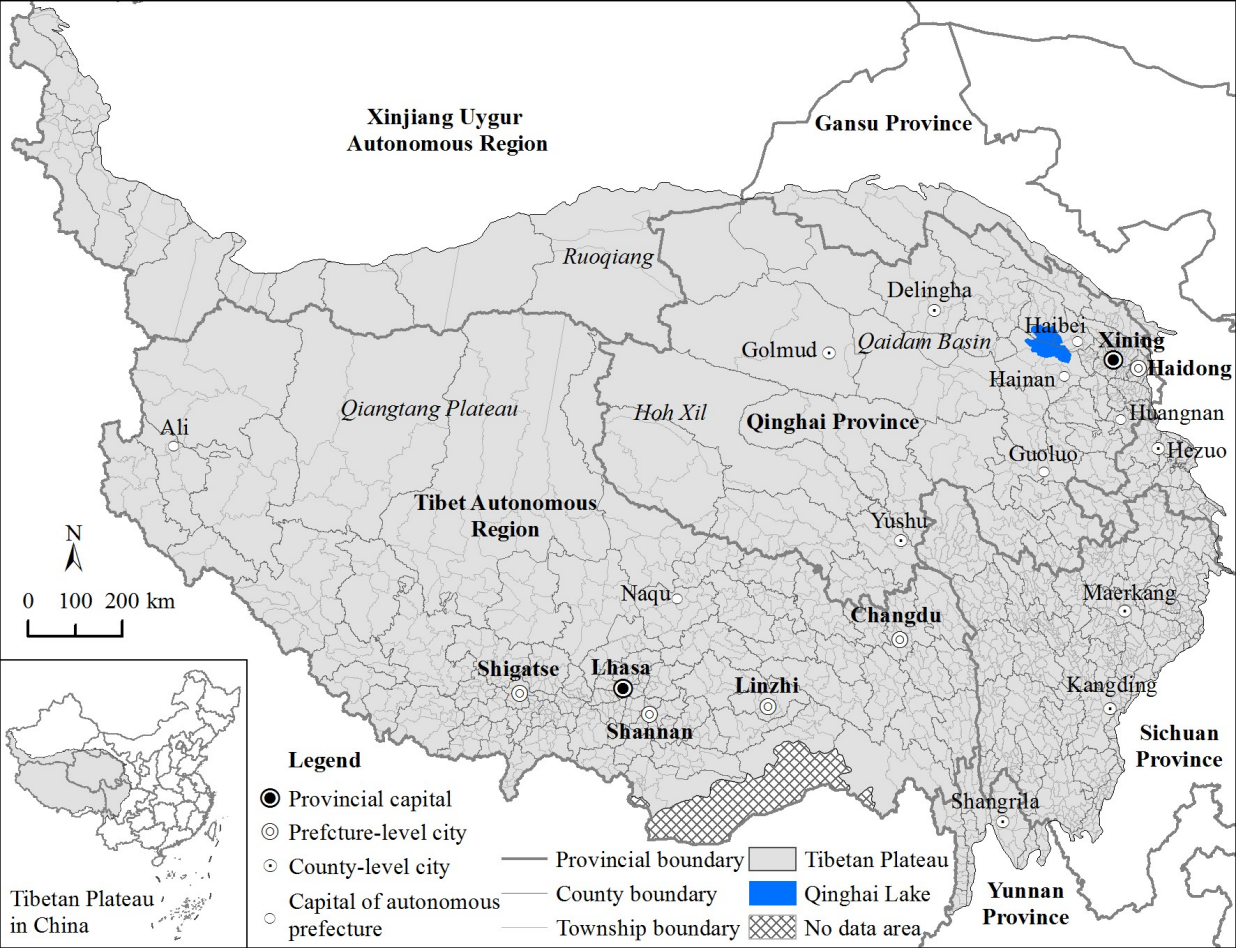
Location of the Tibetan Plateau.

This study takes the Tibetan Plateau as its research area (Fig 1). For a long time, the economic development and traffic conditions of the Tibetan Plateau lagged behind those of other regions in China. Before the peaceful liberation of Tibet, transportation seriously restricted economic development and the possibility of improving herders’ welfare. The construction of main thoroughfares (e.g., Qinghai-Tibet highway, Sichuan-Tibet highway, and Qinghai-Tibet railway) has changed the transport accessibility and economic spatial pattern of the region. The dynamic changes, spatial distribution of transport accessibility and social demand form an important basis for the future development of the Tibetan Plateau.

The Tibetan Plateau is the most unique transportation zone in China and perhaps the world [36]. The development of transportation on the Tibetan Plateau has followed a typical stage-based pattern [30], beginning with “walking and animal power” (before 1954), ordinary roads (1955–1984), railways (after 1984), expressways (after 2001) and HSR (after 2014). Especially after 2000, when China implemented its strategy for the development of its western region (the “Western Development Strategy”) [36, 37], railways were built, and the expressway network expanded throughout the plateau region, greatly improving the region’s transportation network. As such, this paper takes townships as the research unit to discuss the horizontal equity brought by transportation development on the Tibetan Plateau since 1980 and to provide new ideas for transportation construction and sustainable development in pastoral areas.

### Data sources

#### Transportation network data

The transportation and road network data used in this study are based on China’s 1:250,000 database. The road network vector data are obtained from atlases published in 1981, 1991, 2001, 2011 and 2018. To avoid the “island” effect while processing data, creating models and conducting analyses, all road networks in the six provinces that make up the Tibetan Plateau are included. In accordance with the highway design speed specified in the People’s Republic of China Highway Engineering Technical Standards (JTGB 01–2003), with reference to existing research [30], and based on real-life transportation conditions on the Tibetan Plateau and expert interviews, different types of roads in different years are assigned different speed values, and the ArcGIS network analysis tool is used to calculate the shortest reachable time from towns to cities. Neither air nor water transportation are considered in this article.

#### Social demand index

Since the 1980s, scholars have used social demand indexes to explore spatial equity and combined them with the spatial distribution characteristics of social demand and accessibility to reveal transportation equity. Social demand indexes are widely used in research on spatial equity issues [38]. Identifying social demand evaluation indicators is a prerequisite for studying transportation equity. Social demand refers to the potential demand for transportation in a region, which is usually closely related to the population and the economy [39]. This study uses the social demand indicator of township quality (*M*_*i*_), which is based on the permanent population and economic aggregate of each township on the Tibetan Plateau. The formula is as follows:

$$M_{i}=\sqrt{P_{i}\times GDP_{i}}$$ (1)

where *P*_*i*_ is the permanent population of township *i* and *GDP*_*i*_ is the economic aggregate of township *i*. The larger the value of *M*_*i*_ is, the greater the demand for transportation in township *i* is.

Data on the permanent populations of townships on the Tibetan Plateau are mainly derived from the Chinese censuses in 1982, 1990, 2000 and 2010, as well as township permanent resident data reported in the *China County Statistical Yearbook (Township Volume)* from 2018. Missing population data are supplemented by data from county chronicles, yearbooks and statistics from corresponding years or similar years. Economic data on townships on the Tibetan Plateau are derived from the 1-km grid-format GDP dataset for China compiled by the Data Center for Resources and Environmental Science of the Chinese Academy of Sciences [40]. The forecasting grey model GM (1,1) is used to obtain township economic data for 1980 and 1990 (the error with the actual data is less than 5%) [41, 42]. The economic data of administrative divisions of counties and cities and above are derived from the *China County Statistical Yearbook*, *China Statistical Yearbook* and the statistical yearbooks of various provinces and cities.

### Transport accessibility model

Transport accessibility is a quantitative measure of locational transportation advantages or potential [36]. This paper uses the shortest travel time accessibility model to evaluate the shortest time from any point in a region to a city via roads and railways and uses a potential accessibility model to measure a city’s degree of appeal to township social and economic activities [30, 38, 43]. Using the ArcGIS network analysis model, the shortest travel time between town *i* and city *j* is defined as *T*_*ij*_ and is calculated using the following calculation:

$$T_{ij}=Min(t_{ij})i\in (1,2,3,\cdots ,m),j\in (1,2,3,\cdots ,n)$$ (2)

where *T*_*ij*_ is the time accessibility value of township *i*. The smaller the *T*_*ij*_ value is, the better the accessibility is and convenient the township’s transportation is. The term *t*_*ij*_ denotes the travel time between township *i* and city *j* (minimum values are used in this study).

Potential accessibility combines transportation costs and the scale of social activities and is often used to measure location conditions, providing a full balance between complexity and interpretability [20]. Potential accessibility is defined as the spatial interaction between towns and cities. Considering internal potential [44], the formula is as follows:

$$A(P)=\frac{P_{i}}{T_{ii}^{\alpha }}+\sum_{j=1}^{n}\frac{P_{j}}{T_{ij}^{\alpha }}$$ (3)

where *A(P)* is the potential accessibility value (the larger this value is, the better the location conditions are); *P*_*i*_ is the population of township *i*; *T*_*ii*_ is the average travel time within that township; *P*_*j*_ is the population of city *j*; and *α* represents the coefficient of transportation friction (*α* is 1 in this study) [30, 45]. For convenience, the following formula is usually used:

Tii=(Ai/π)2s (4)

where *A*_*i*_ is the area of township *i* and *s* is the assumed internal driving speed of 6 km/h.

The accessibility calculation model is used to analyse the shortest travel time from various parts of the Tibetan Plateau to cities. It is combined with population data to obtain the economic potential between the target city and township, and inverse distance weighted interpolation is used to obtain the spatial pattern of the Tibetan Plateau’s transport accessibility and its evolution. In addition, the spatial statistics function is used to calculate transportation accessibility of the 1,972 township divisions of the Tibetan Plateau.

### Equity measurement method

#### Coefficient of variation

The construction and development of transportation infrastructure undoubtedly improves accessibility efficiency, but it is also true that townships located along important transportation routes benefit more from accessibility than townships farther away from those routes. As a result, previous studies have tended to use the coefficient of variation and standardised values to measure and assess the spatial equity of accessibility [26, 46, 47]. The general formula for calculating transportation equity is as follows:

$$CV=\frac{1}{T_{ave}}{\left(\frac{1}{n}\sum_{i=1}^{n}{\left(T_{i}-T_{ave}\right)}^{2}\right)}^{1/2},i=1,2,\cdots ,n$$ (5)

where *CV* is the coefficient of variation; *T*_*ave*_ represents the average accessibility value; *n* represents the number of townships; and *T*_*i*_ is the accessibility value of town *i*. The higher the *CV* value is, the lower the level of equity and the greater the difference in the level of accessibility between townships are, indicating that transportation development has had a negative impact on equity. Conversely, a low *CV* value indicates a positive impact on equity and a more equitable spatial distribution of accessibility.

#### Gini coefficient

The Gini coefficient and Lorenz curve are the earliest tools used to analyse the income distribution of a population, but they have been used to measure various equity issues in the field of economics, such as income and transportation [10, 48]. The Lorenz curve is an intuitive representation of a cumulative distribution function, and the Gini coefficient is a mathematical measure of the overall degree of inequality. The following formula is used [48, 49]:

$$G_{k}=abs\left(1-\sum_{k=1}^{n}\left(X_{k}-X_{k-1})(Y_{k}+Y_{k-1}\right)\right)$$ (6)

where *G*_*k*_ is the Gini coefficient for year *k*; *X*_*k*_ is the cumulative proportion of the social demand variables; *Y*_*k*_ is the cumulative proportion of the accessibility variables; and *k* is the year. In this study, the township social demand index is divided into 10 levels (representing deciles), from high to low, to obtain the accessibility ratio of each group and calculate the Gini coefficient.

### Spatial autocorrelation

Spatial autocorrelation refers to the degree of correlation between a certain geographic phenomenon or a certain attribute value in a certain spatial area and the same phenomenon or attribute value in an adjacent spatial area. It can be divided into global and local spatial autocorrelations. Global spatial autocorrelation is the study of the spatial distribution of a certain attribute value in the entire research area. Local spatial autocorrelation can detect spatial differences caused by spatial autocorrelation and is used to analyse the specific distribution of the aggregation area in the entire research area.

Bivariate spatial autocorrelation is used to analyse autocorrelation between the target variable and another variable in the spatial region. The usual indicator is the bivariate Moran’s I index, with a value range of [−1, 1], and the Z value test is performed. If Moran’s I>0 and Z≥1.96, it means that the clustering of the target variable in the space is positively correlated with the other variable. If Moran’s I<0 and Z≤-1.96, it means that the clustering of the target variable in the space has a negative correlation with the other variable. If Moran’s I = 0, it means that the two variables have no correlation. In this paper, bivariate local spatial autocorrelation analysis is used to analyse spatial matching between the social demand of a township and transport accessibility, allowing the visualisation of the spatial pattern of local differences.

The calculation formula of the bivariate Moran’s I is as follows [50]:

$$I_{i}=\frac{\sum_{i}\left(\sum_{j}w_{ij}y_{j}\times x_{i}\right)}{\sum_{i}x_{i}^{2}}$$ (7)

where *x*_*i*_ and *x*_*j*_ are the attribute values of towns *i* and *j*, respectively, and *w*_*ij*_ is the spatial weights matrix, indicating adjacency between towns *i* and *j* (if they are adjacent, *w* = 1; if not, *w* = 0).

## Evolution and equity of transport accessibility

### Transport accessibility evolution and characteristics

Cities are the development poles on the Tibetan Plateau. They are important nodes of population, economy and transportation [30], and they have a strong cohesive effect regionally. As a result, the evolution and characteristics of transport accessibility between townships and cities have had an important impact on the social and economic development of townships in the vast and sparsely populated plateau region.

#### Time accessibility

**Fig 2:**
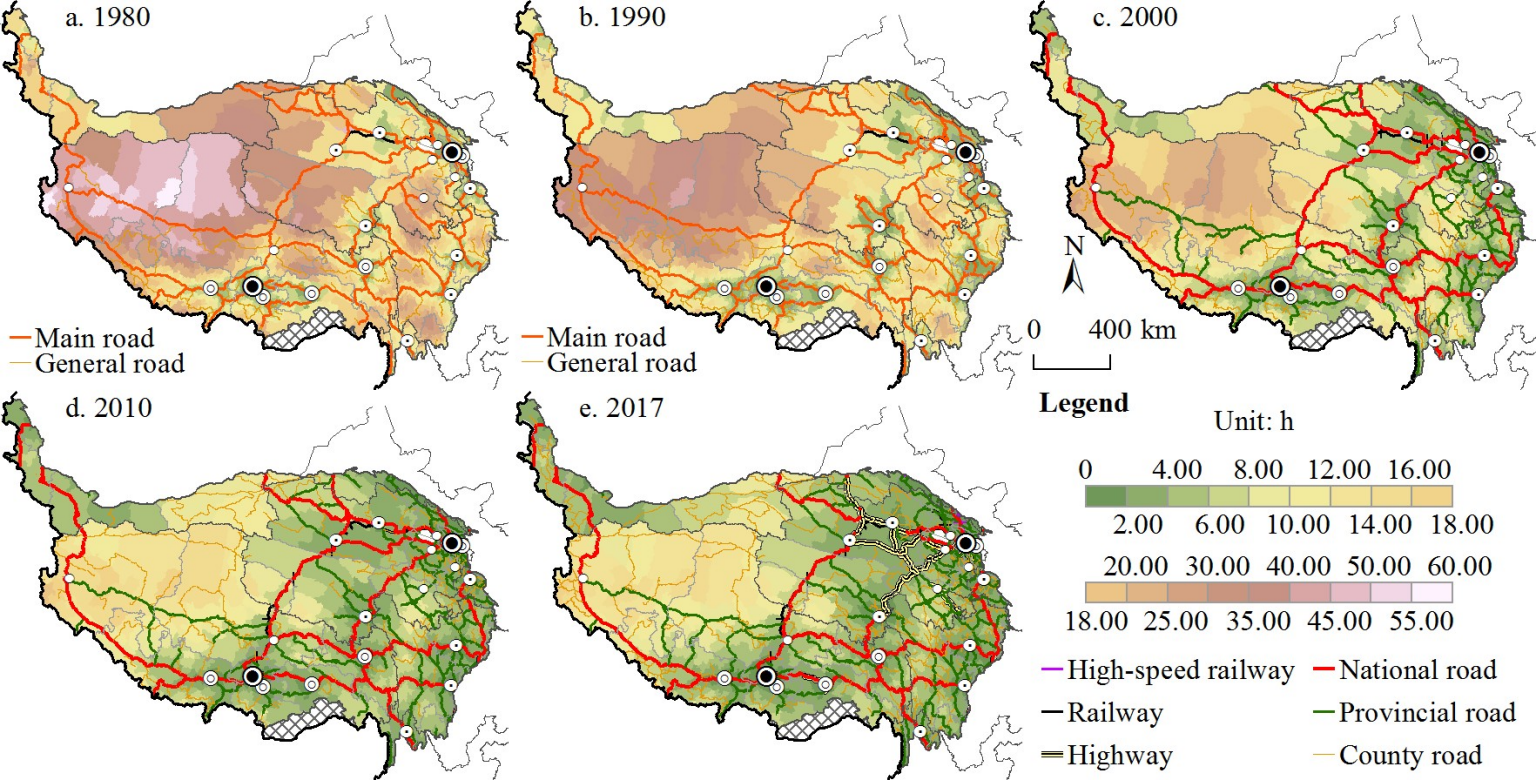
Spatial patterns and changes in time accessibility between townships and major cities from 1980 to 2017.

The vast and sparsely populated Tibetan Plateau has few cities. Provincial capitals, prefecture-level cities and county-level cities constitute the region’s major urban areas. They are mainly located in the northeast, east and south regions of the plateau, while cities on the periphery of the plateau are mainly found in the Sichuan Basin and the Hexi Corridor (Fig 1). As a result, the distribution pattern of time accessibility shows that Ali Prefecture, Qiangtang Plateau, the Hoh Xil region and Ruoqiang County have long journey times and poor accessibility to cities (Fig 2). The main reason for this result is the lack of cities in these areas and the small number and low density of major traffic arteries. With the development of the transportation network, especially as a result of China’s Western Development Strategy, the accessibility level has greatly improved, and the time cost from townships to cities has shortened significantly, with the longest time reduced from 59.1 hours in 1980 to 17.69 hours in 2017 and the average time shortened from 13.05 hours to 3.41 hours (Table 1).

**Table 1:** Transport accessibility between townships and major cities.

Year	Access to the nearest city (hours)			Potential accessibility		
	Min.	Max.	Average	Min.	Max.	Average
**1980**	0.35	59.10	13.05	26.31	889.41	101.62
**1990**	0.16	43.59	9.37	48.57	1352.98	200.72
**2000**	0.10	27.31	5.84	80.38	2475.01	342.85
**2010**	0.07	19.43	3.81	137.44	2862.24	589.00
**2017**	0.06	17.69	3.41	151.85	3288.06	659.60

Townships with good journey time accessibility are concentrated in the vicinity of cities and along important traffic arteries, such as the Hexi Corridor, Huangshui River valley, the “One River and Two Streams” Region in Tibet (the Yarlung Zangbo River, Nianchu River and Lhasa River region), Qaidam Basin (Delingha and Golmud) and the edge of the Sichuan Basin, which is closely linked to the spatial distribution of cities. The townships with the greatest change in accessibility levels are mainly those with large initial journey times that are far from cities and traffic arteries. This is consistent with the conclusions of previous studies [36].

#### Potential accessibility

**Fig 3:**
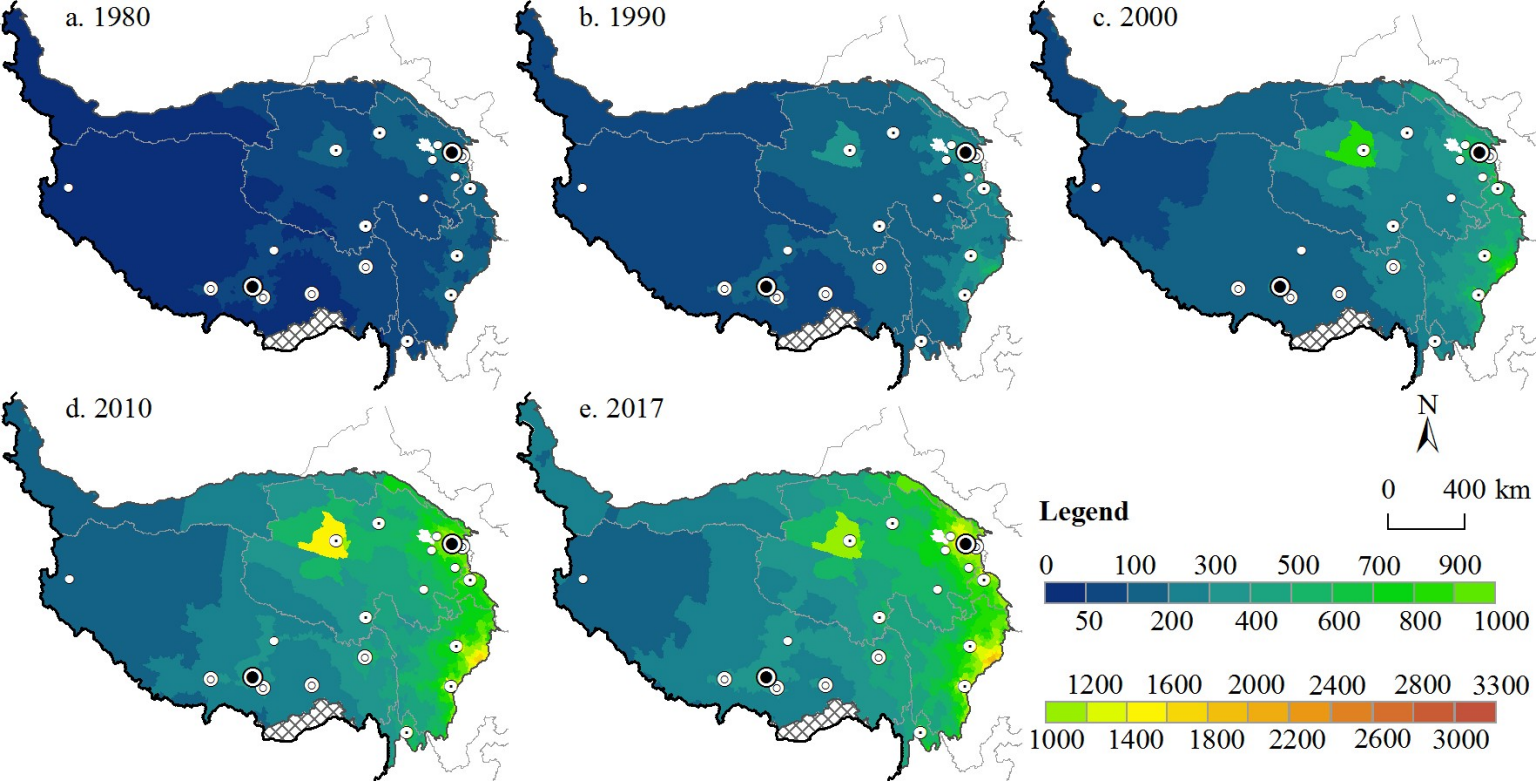
Spatial patterns and changes in potential accessibility at the township scale.

Unlike time accessibility, potential accessibility combines transportation costs and the scale of activity, and it is often used to measure changes in location conditions brought about by the development of transportation infrastructure [30]. Figs 2 and 3 show that the potential accessibility and time accessibility of the Tibetan Plateau have similar spatial patterns. The townships in the Ali Prefecture, Qiangtang Plateau, Hoh Xil region and Ruoqiang County are far from major cities, so they have lower potential accessibility values and poor accessibility, while the potential accessibility values of townships in surrounding cities are high. With the construction of transportation infrastructure, average potential accessibility is increasing year after year, and it increased approximately 5.5-fold between 1980 and 2017, with minimum potential accessibility increasing from 26.31 to 151.85. Townships with good potential accessibility were mainly located in the eastern part of the Tibetan Plateau or near the hinterland cities of Lhasa, Golmud and Changdu. Most townships in the Tibet Autonomous Region had relatively poor potential accessibility.

**Fig 4:**
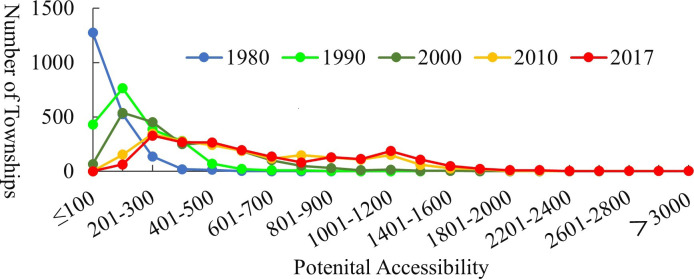
Potential accessibility distribution and statistics.

Fig 4 shows that the number of townships with low potential accessibility values decreased over the study period, and the number of townships with high potential accessibility scores increased. Townships with a potential accessibility score of more than 100 in 1980 gradually evolved into areas with better accessibility. Especially after 2000, the proportion of towns with low accessibility levels (≤300) fell below 20%.

There are three primary reasons for the formation and evolution of this spatial pattern. First, the populations of cities on the Tibetan Plateau, such as Lhasa and Linzhi (Nyingchi), are relatively small, while cities on the periphery of the Tibetan Plateau, such as Chengdu, Deyang and Lanzhou, have larger populations [49], so they have a strong appeal to townships in the plateau region, which has led to poor potential accessibility in most parts of Tibet. The second reason is the rapid transportation network, consisting of high-speed railways and vehicle expressways, between the plateau and surrounding areas, which are mainly distributed in the Huangshui Valley, Sichuan Basin and Hexi Corridor [30]. The expressway network within the plateau is still in the construction and expansion stage, which means that the time cost from most townships to Chengdu, Lanzhou and other large cities is still relatively high. Third, since the implementation of the Western Development Strategy in 2000, cities on the Tibetan Plateau have experienced rapid social and economic development. In 2017, the economic volume and population of urban districts accounted for approximately 50% and 30% of the regional totals, respectively [51], making these districts more economically appealing to surrounding townships.

### Transportation equity

The coefficient of variation (CV) of transport accessibility can be used to evaluate transportation equity. Table 2 shows that between 1980 and 2017, the time accessibility CV and potential accessibility CV changed by 0.0121 and 0.0265, respectively, indicating that the difference in transport accessibility between townships increased and that the difference in the appeal of cities and townships widened. Specifically, the time accessibility CV decreased slightly between 1980 and 2000, from 0.7287 to 0.7169, and then rose to 0.7408 in 2017; the potential accessibility CV changed slowly at first and then quickly, increasing from 0.6139 in 1980, to 0.6246 in 2000, and to 0.6404 in 2017. This increase in the CV value indicates that the development of the transportation network reduced the spatial equity of township accessibility, which had a negative impact. The overall CV value of transport accessibility showed an increasing trend, indicating spatial inequality in the time accessibility from towns to cities in the region. This inequality was more significant following the implementation of the Western Development Strategy. The reason for this result is that before 2000, the Tibetan Plateau mainly had ordinary roads, so it suffered from technical constraints; and railways did not yet fully exert an influence on the accessibility of townships. As a result of railway construction and speed increases as well as the expansion of high-grade roads after 2000, the pattern of accessibility changed. As a corridor effect emerged along important traffic arteries, accessibility equity decreased.

**Table 2:** Global coefficient of variation of transportation equity and the Gini coefficient of transport accessibility on the Tibetan Plateau.

Year		1980	1990	2000	2010	2017
**Coefficient of variation**	Time accessibility	0.7287	0.7254	0.7169	0.7315	0.7408
	Potential accessibility	0.6139	0.6191	0.6246	0.6330	0.6404
**Gini coefficient**	Time accessibility	0.1753	0.1621	0.1615	0.1714	0.1688
	Potential accessibility	0.1366	0.1540	0.1547	0.1911	0.1285

The Lorenz curve of accessibility over the years is used to calculate the Gini coefficient of transport accessibility on the Tibetan Plateau (Table 2). The Gini coefficient of time accessibility in the region fluctuated, rising from 0.1688 in 1980 to 0.1753 in 1990, then dropping to 0.1621 in 2000 and 0.1615 in 2010, and then increasing again to 0.1714 in 2017. The Gini coefficient of potential accessibility is consistent with the trend of potential accessibility CV (both increasing), increasing from 0.1285 in 1980 to 0.1540 in 2010 and to 0.1911 in 2017, which indicates that the degree of transportation equity on the Tibetan Plateau decreased. From this analysis, time accessibility only reflects the time cost from townships to cities and does not include the socioeconomic development brought about by transportation improvements, whereas potential accessibility, which combines economic development and transportation improvements, is more suitable for measuring transportation equity.

## Township social demand and transport accessibility

### Changing pattern of the transportation social demand index

**Fig 5:**
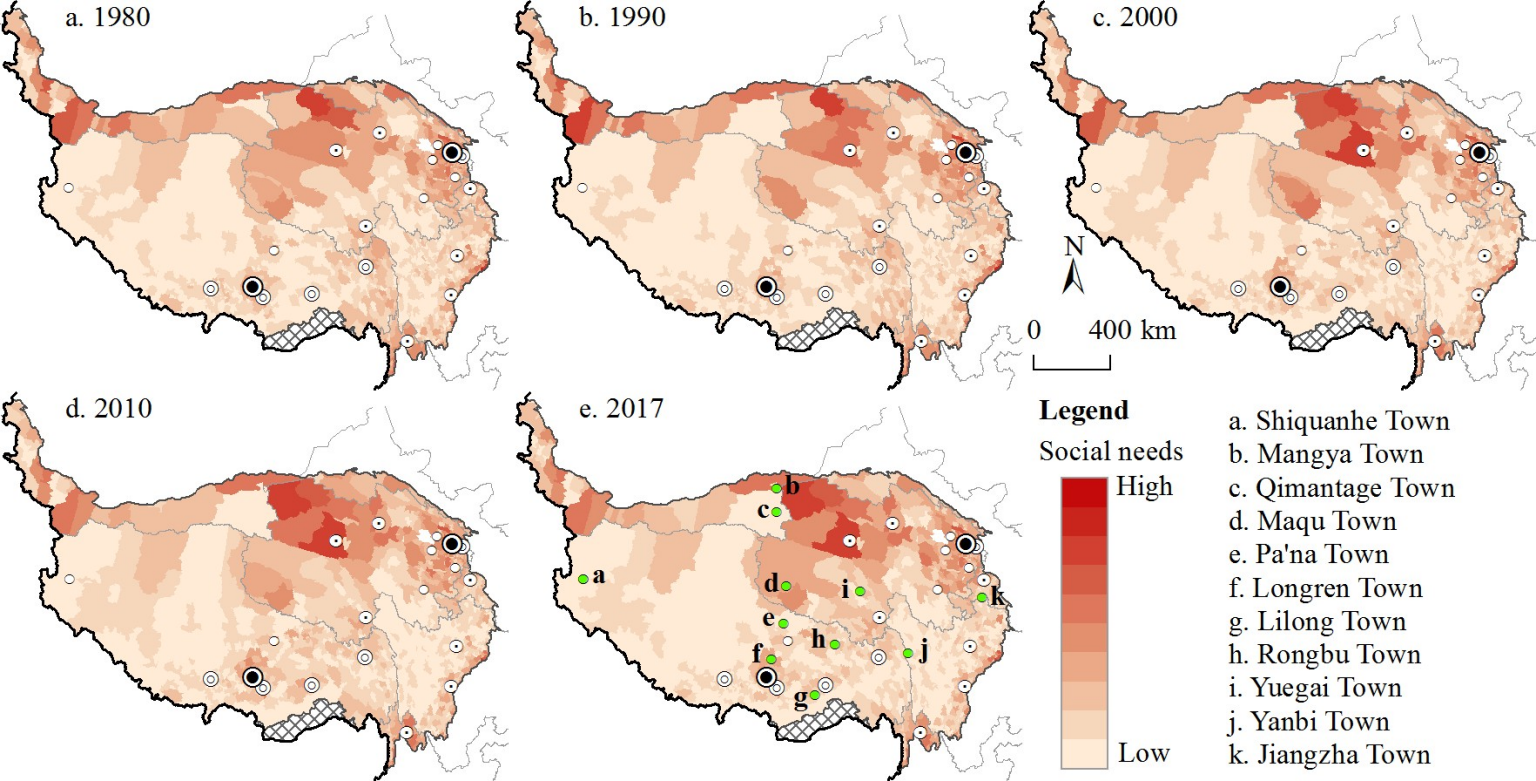
Distribution of social demand deciles from 1980 to 2017.

Fig 5 shows the spatial distribution of the deciles of the social demand index for townships on the Tibetan Plateau from 1980 to 2017. The lightest colour represents the decile of townships with the lowest social demand, that is, the township divisions with the smallest population and economic aggregate in the region. The darker the colour is, the higher the social demand of townships is, which means they need greater transport accessibility. Townships with low social demand index scores are mainly distributed in Ali, the Qiangtang Plateau, Shigatse, Guoluo, Aba, Ganzi and other regions, as well as parts of southern Tibet. Townships with higher social demand index scores are concentrated in the Hehuang Valley (Xining and Haidong), on the eastern edge of the Tibetan Plateau, in northern plateau areas, the Qaidam Basin, and in cities and their surrounding areas. Different from the spatial distribution of the population on the Tibetan Plateau [52], the social demand index is more affected by the economic distribution.

Influenced by factors such as economic development and population change, the overall social demand of townships on the Tibetan Plateau has shown an upward trend, with the average score increasing from 0.0758 in 1980 to 0.1598 in 1990 and to 0.3472 in 2000. After the Western Development Strategy began in 2000, the average score increased to 0.7846 and then 1.1246. This means that social demand index scores have doubled every decade. The townships with the largest increase in social demand index scores are mainly municipal districts and townships, central cities and townships with relatively large populations, such as Chengguan District (Lhasa), Guolemude Town (Golmud), Qiaotou Town (Datong County), and resource development towns, such as Huatugou and Lenghu. Looking at the decile rankings of townships from 1980 to 2017, the townships Yuegai, Lilong, Maqu, Rongbu, Longren and Shiquanhe increased the most, indicating that they experienced relatively high population growth or faster economic development, which increased the demand for transportation. The townships of Qimantage, Yanbi, Jiangzha, Pa’na and Mangya declined the most in the rankings, indicating that the populations or economies of these townships decreased significantly, with the majority affected by the former [53]. For example, the total population of Mangya decreased from 13,000 in 1980 to 5,500 in 2017, and the total population of Qimantage decreased from 1,100 to less than 100.

### Changing accessibility based on the social demand index

To further analyse the relationship between transport accessibility and social demand at the township scale on the Tibetan Plateau, this study uses Z-score normalisation to normalise relevant indicators. Table 3 shows differences in social demand index scores, time accessibility, and potential accessibility of townships in different deciles from 1980 to 2017. Overall, there was a steadily narrowing trend, with changes of -0.0096, 0.1231 and -0.0597 and changes in median values of -0.0054, 0.1649 and -0.1055, respectively. To a certain extent, this reflects the development of transportation infrastructure, which reduced the average travel time between townships and cities and improved the location conditions of towns. In addition, the gap between social demand index scores and potential accessibility first declined and then rose between 2000 and 2017. In other words, in the first decade of the Western Development Strategy (2000 to 2010), there was a decline, and after 2010, there was an increase. This phenomenon was closely related to the concentration of population and economic factors in county towns and cities, which led to a widening gap in the level of economic development between townships.

**Table 3:** Normalised values of the dataset.

Indicator	Year	Max.	Min.	Range	Median
**Social demand index**	1980	2.6803	-0.6577	3.3380	-0.3641
	1990	2.6890	-0.6428	3.3318	-0.3652
	2000	2.6680	-0.6611	3.3291	-0.3707
	2010	2.6695	-0.6571	3.3265	-0.3720
	2017	2.6887	-0.6398	3.3284	-0.3695
**Time accessibility**	1980	1.5936	-1.5310	3.1246	-0.1501
	1990	1.4954	-1.5415	3.0369	-0.1585
	2000	1.4702	-1.5977	3.0680	-0.0445
	2010	1.4651	-1.6038	3.0689	0.0682
	2017	1.4633	-1.5383	3.0015	0.0148
**Potential accessibility**	1980	2.1995	-1.0868	3.2863	-0.2550
	1990	2.1491	-1.1715	3.3206	-0.2398
	2000	2.2781	-1.0458	3.3239	-0.3582
	2010	2.1803	-1.0165	3.1968	-0.3907
	2017	2.1805	-1.0461	3.2266	-0.3605

**Fig 6:**
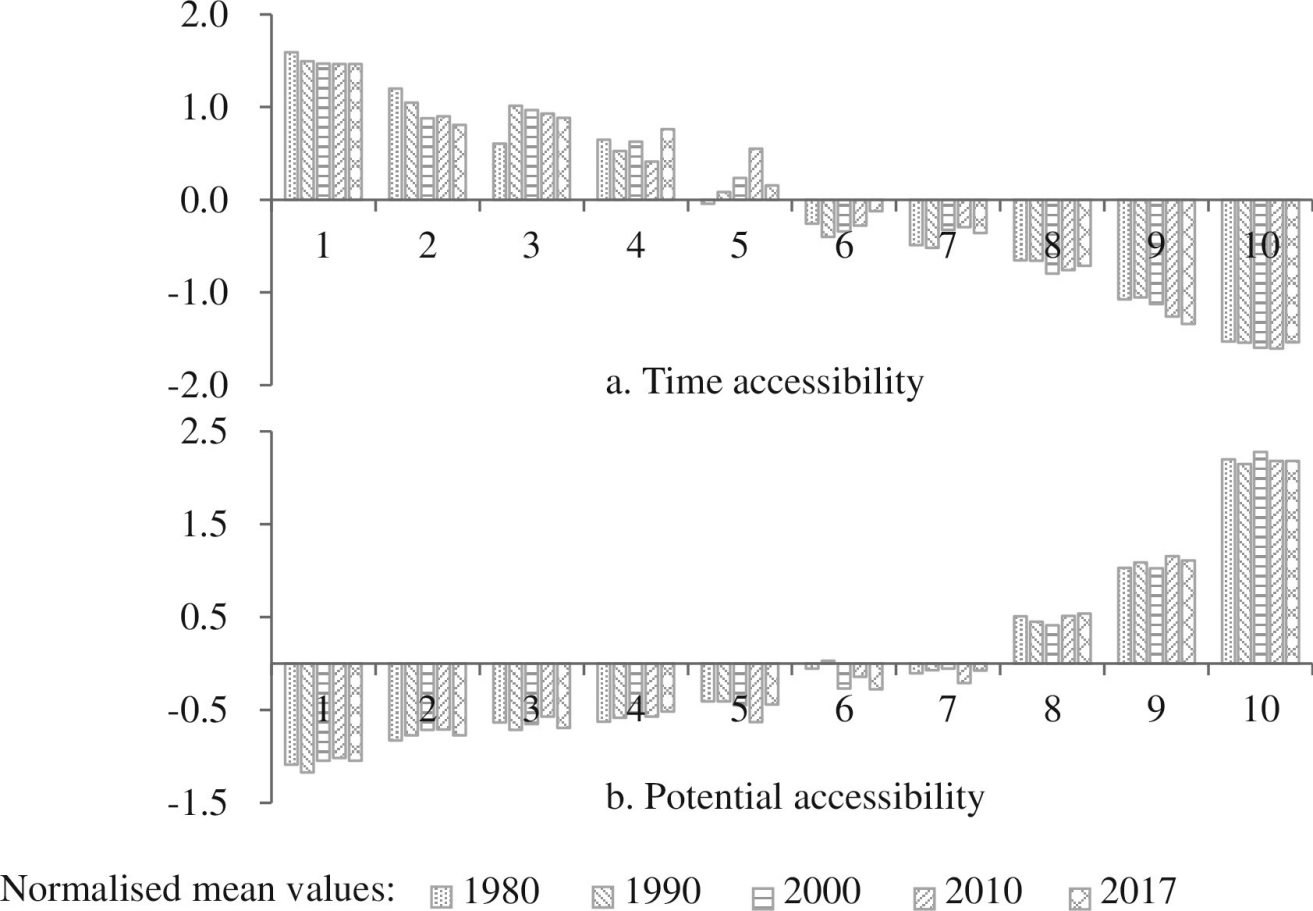
Transport accessibility represented by social demand index deciles.

Having analysed the distribution of accessibility based on the deciles of the social demand index scores for townships on the Tibetan Plateau from 1980 to 2017, the results in Fig 6 show that a smaller number on the horizontal axis equates to lower social demand. Time accessibility is an inverse indicator: a larger value equates to a lower level of accessibility, and a decrease in value means that the transportation level increases. The opposite is true of potential accessibility, which is a positive indicator. It can be calculated from Fig 6A that in the past 37 years, the mean time accessibility of townships in deciles 1, 2, 3, 9 and 10 changed by -0.0073, -0.2664, -0.0589, -0.3148 and -0.1304, respectively. This shows that compared with townships in other deciles, transportation development is more conducive to shortening the travel time from townships to cities in areas with the lowest and highest populations and economic aggregates. The deciles of potential accessibility (Fig 6B) indicate that the benefits of transportation improvement in deciles 1, 2, 4, 8 and 9 were relatively significant, with mean values increasing by 0.0407, 0.0538, 0.1095, 0.0270 and 0.0815, respectively, while the potential accessibility of townships in other deciles declined. Hampered by natural conditions and other factors, transportation development on the Tibetan Plateau lagged behind that of other regions for a long time and became an important factor restricting social and economic development. Following the promotion of national policies and infrastructure construction, such as the Western Development Strategy, transportation facilities on the Tibetan Plateau improved greatly. This has played an important role in promoting the socioeconomic development of the region and improving people’s standard of living.

By analysing changes in social demand and accessibility of township divisions on the Tibetan Plateau, we can see that between 1980 and 2017, the social demand of townships showed an increasing trend in all deciles, with changes in demand gradually increasing, and changes in potential accessibility gradually decreasing, while there were no significant discrepancies in time accessibility changes. Taking the township deciles with the lowest and highest social demand (first and tenth deciles) as examples, the social demand in 2017 was 12.86 times and 15.47 times higher than in 1980, respectively, and potential accessibility increased 6.66 and 4.89 times, respectively. Travel time accessibility was shortened by 25.55% in the first decile and 25.38% in the tenth decile. This reveals that time accessibility has certain limitations when discussing transportation equity and that it needs to be combined with other accessibility indicators. In addition, transportation development can greatly improve the accessibility of areas with insufficient socioeconomic development and constantly promote regional transportation equity.

### Spatial relationship between social demand and transport accessibility

In view of the limitations of using time accessibility to discuss equity, this study uses potential accessibility to analyse the spatial relationship between social demand and transportation of townships on the Tibetan Plateau. A bivariate local spatial autocorrelation analysis of the township social demand index and potential accessibility on the Tibetan Plateau shows that Moran’s I values from 1980 to 2017 are all >0 and that the p-values are all <0.01 (Table 4), which reveals a positive correlation between the spatial agglomeration of social demand in townships and the distribution of potential accessibility.

**Table 4:** A bivariate local spatial autocorrelation analysis of township social demand and potential accessibility.

	1980	1990	2000	2010	2017
**Moran’s I**	0.4245	0.3755	0.4024	0.3816	0.3866
***Z* value**	35.2648	34.6704	35.0853	33.6749	34.5368
***p* value**	<0.01	<0.01	<0.01	<0.01	<0.01

There are four types of clusters: low-low clusters, low-high clusters, high-low clusters and high-high clusters. Low-low clusters and high-high clusters indicate that the matching degree between social demand and transport accessibility is good. Low-high clusters indicate that the level of transportation development exceeds economic development, while high-low clusters mean that traffic cannot meet the needs of economic development.

**Fig 7:**
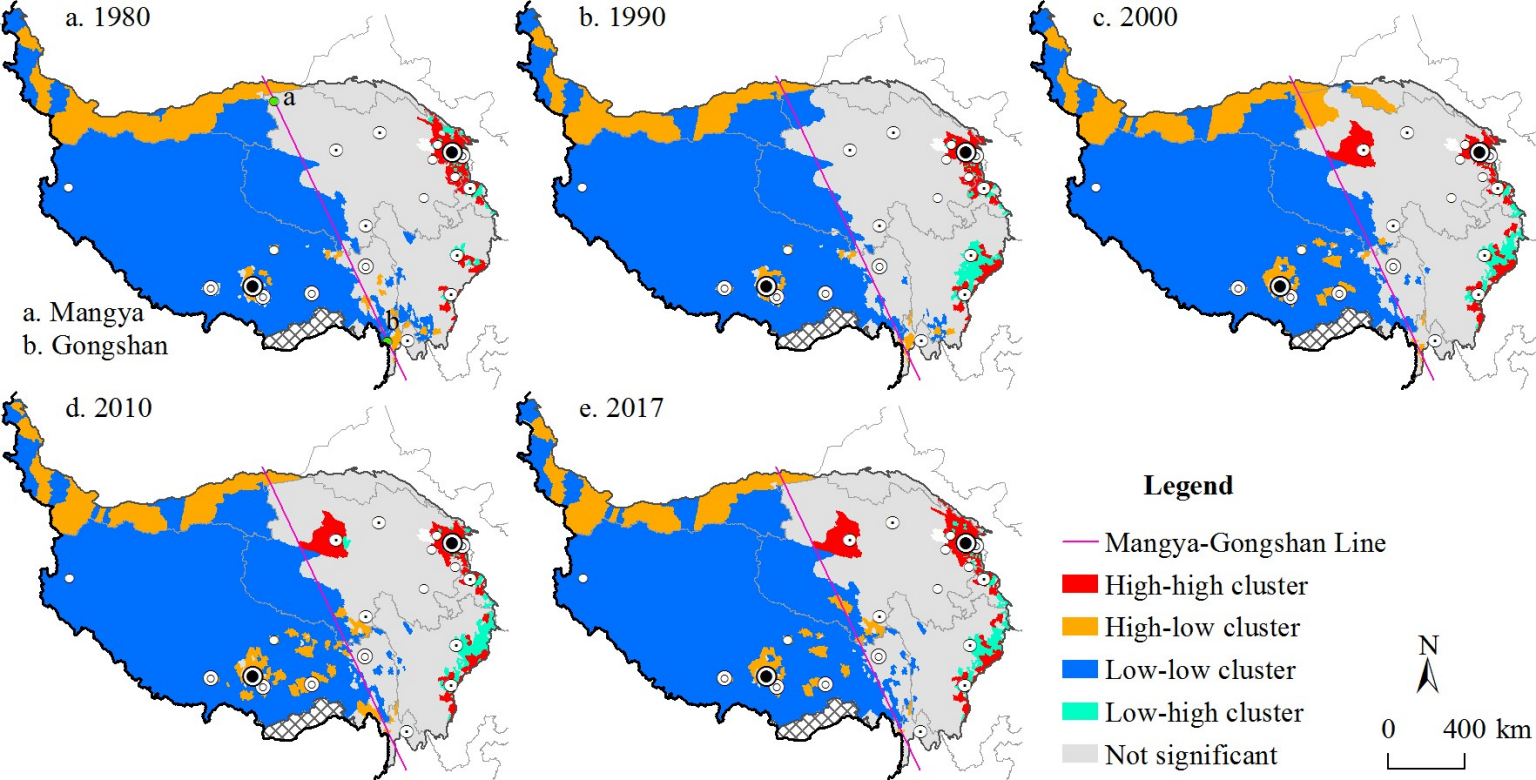
Bivariate local spatial autocorrelation clustering of township social demand and potential accessibility.

Between 1980 and 2017, aggregations of types of social demand and potential accessibility of townships were relatively stable in terms of spatial distribution, with strong distribution regularity. As shown in Fig 7, low-low clusters were the main type, followed by high-high clusters. High-high clusters were concentrated in the north-eastern plateau (Xining and Hezuo), around Kangding and near Golmud. High-high clusters have high social demand and good transportation conditions. In 2000, when China implemented the Western Development Strategy, resources were further developed in the Qaidam Basin, and Golmud and surrounding townships changed to high-high cluster townships. The high-low clusters are townships with high social demand and poor location conditions, indicating that transportation development cannot meet the social and economic transportation needs of the township. High-low township clusters are mainly distributed on the northern edge of the Tibetan Plateau (in Xinjiang) and in and around townships of municipal and county governments in the hinterland of the plateau (e.g., Lhasa, Linzhi and Nangqian).

Low-low clusters, which have low social demand and poor transportation links, are concentrated west of the Mangya-Gongshan Line. Their general characteristics include small populations, economies dominated by animal husbandry, high-elevation locations, fragile environments, long distances from cities, and lacking rapid transportation methods connecting them with cities. Township clusters of low social demand and high potential accessibility (low-high clusters) are mainly distributed in Hezuo, Maerkang and Kangding.

As seen from this analysis, there emerged a regional pattern in the spatial autocorrelation clustering of social demand and transport accessibility of townships on the Tibetan Plateau between 1980 and 2017.

Townships with high social demand and high accessibility were concentrated in the Hehuang Valley (Xining and Haidong) as well as near Golmud, Hezuo and Kangding. Low-low clusters were concentrated west of the Mangya-Gongshan Line, and low-high clusters were mainly distributed around Maerkang, Hezuo and Kangding.

Clusters with high social demand and insufficient transportation (high-low clusters) were located in two main areas. The first was along the northern edge of the Tibetan Plateau. Most of these areas rely on oasis agriculture, animal husbandry and resource development, so they have high transportation demands, but they are far from cities; their existing transportation includes ordinary highways (National Highway G315), and they lack railways and expressways. The second area of high-low clusters was concentrated in and around the cities of Lhasa, Linzhi, Naqu and Nangqian. These areas are the main heavily populated urbanised areas in the plateau’s hinterland.

## Conclusion and discussion

By considering both accessibility and social needs, this article analysed the temporal and spatial characteristics of transport accessibility and spatial equity on the Tibetan Plateau at the township scale. First, time accessibility and potential accessibility were examined to explain spatial changes in and characteristics of transport accessibility in the plateau region; second, the coefficient of variation and Gini coefficient of accessibility were combined with a social demand index to explain spatial equity; and finally, a township social demand index was constructed using the factor of permanent population and economic aggregate, and the spatial relationship between social demand and transport accessibility was discussed. The main conclusions are as follows.

1. Transportation development will undoubtedly improve accessibility, which is consistent with the existing research conclusions [20, 30, 36, 46]. The construction of transportation infrastructure has greatly improved accessibility on the Tibetan Plateau, reduced transportation costs, and promoted social and economic exchanges and flows of people and commodities in the townships of the plateau region. Transportation development has reshaped the spatial pattern of accessibility, forming areas of high-level accessibility with important cities as nodes and major traffic arteries as axes and low accessibility in townships in areas such as Ali Prefecture, Qiangtang Plateau, Hoh Xil region and Ruoqiang County. Over the past 37 years, the average travel time from townships to cities has been shortened from 13.05 hours to 3.41 hours, so the time cost has been greatly reduced, and the average potential accessibility has increased each year by approximately 5.5-fold in total.
2. Transportation development leads to inequality in the spatial distribution of accessibility [7], and the development of the transportation network has magnified the difference in the spatial distribution of accessibility on the Tibetan Plateau. The coefficient of variation of transport accessibility and the Gini coefficient both increased slightly. Between 1980 and 2017, differences in transport accessibility between townships widened, and differences in the appeal of cities to townships also increased. Using the Gini coefficient, the coefficient of variation of potential accessibility changed greatly following implementation of the Western Development Strategy, indicating that with the construction of high-grade railways and expressways, the pattern of accessibility changed, and a corridor effect emerged along major traffic arteries. This corridor effect caused a decrease in transport accessibility equity among townships on the Tibetan Plateau.
3. Social demand index scores trended upwards, and there were regional characteristics of spatial distribution. This distribution feature is more affected by the economy than other features are. Social demand for transportation among townships in the plateau region is doubling every ten years, with the average index score increasing from 0.0758 in 1980 to 0.1598 in 1990 and to 0.3472 in 2000. After the start of the Western Development Strategy in 2000, the average index score increased further to 0.7846 and then to 1.1246. In terms of spatial distribution, townships with high social demand index scores were mainly located in the Hehuang Valley, along the eastern edge of the plateau, in the Qaidam Basin, in and around cities, and along the Qinghai-Tibet Railway. The main reason for declining social demand index scores was decreasing permanent populations of townships.
4. Using the decile system, the gaps among the social demand index scores, time accessibility and potential accessibility were observed to narrow. Transport accessibility represented by social demand index deciles showed that transportation development brought greater benefits to townships in the lowest and highest deciles. Between 1980 and 2017, the social demand of townships showed an increasing trend in all deciles, with changes in demand gradually increasing and changes in potential accessibility gradually decreasing, while there were no significant discrepancies in time accessibility changes.
5. There was a significant positive spatial relationship between social demand and potential accessibility (location conditions), and clusters had a highly evident geographical distribution. From the matching degree of traffic and social demand, high-high clusters (townships with high social demand and good location conditions) are concentrated in the Hehuang Valley and near the cities of Golmud, Hezuo and Kangding; high-low cluster types are located along the northern edge of the plateau and in and around the cities of Lhasa, Linzhi and Naqu; low-low township clusters are concentrated west of the Mangya-Gongshan line; and low-high clusters are mainly distributed around Maerkang, Hezuo and Kangding.

We noticed that the towns with a good matching degree of transport accessibility and social demand are mainly distributed around Xining, Golmud, Hezuo and other cities on the Tibetan Plateau (high transport accessibility and high social demand) and west of the Mangya-Gongshan line (low transport accessibility and low social demand). Transport accessibility and social demand follow clear distribution law. China’s urbanisation process and ecological migration policy promote the population migration of the Qiangtang Plateau (northern Tibet) and Hoh Xil (southwestern Qinghai) to Golmud, Lhasa and other cities. This leads to further reductions in the demand for transportation in areas with low social demand, such as the Qiangtang Plateau and Hoh Xil. The construction of major transportation infrastructure (e.g., Lhasa-Linzhi railway, Beijing-Tibet expressway, Sichuan-Tibet railway, Southern Xinjiang railway and Xingjiang-Tibet railway) meets the transportation service demand of high-low clusters in Lhasa and southern Xinjiang and may turn into high-high clusters. The decrease in population and the development of transportation promote the transformation of low-low type towns into low-high type towns.

This focus of this study was the relationship between accessibility and social demand. It analysed and discussed the impact of transportation development on accessibility and spatial equity on the Tibetan Plateau. Social demand indexes in existing research mainly focus on urban areas and different groups of people. Concentrating on townships in extremely impoverished areas with fragile environments, this study selected the factors of population and economy to construct a social demand index and combined it with the spatial distribution of time accessibility and potential accessibility to further analyse the equity of the spatial distribution of transportation. It can serve as an important reference for research on regional transport accessibility and spatial equity. Further development of transportation on the Tibetan Plateau (e.g., Sichuan-Tibet railway, Yunnan-Tibet railway, and Xinjiang-Tibet railway) will change the regional distribution of transportation and social demand and even lead to exceeding expectations of transportation development. In view of Tibet’s important position in geopolitics, what kind of geopolitical impact will moving ahead of schedule with transportation on the Tibetan Plateau have on the surrounding areas? This will be a topic worth exploring.

## Supporting information

S1 Data (RAR)
